# The isotype repertoire of antibodies against novel UH-RA peptides in rheumatoid arthritis

**DOI:** 10.1186/s13075-016-1030-1

**Published:** 2016-06-07

**Authors:** Liesbeth M. De Winter, Piet Geusens, Jan Lenaerts, Johan Vanhoof, Piet Stinissen, Veerle Somers

**Affiliations:** Biomedical Research Institute, Hasselt University, and School of Life Sciences, transnational University Limburg, Martelarenlaan 42, 3500 Hasselt, Belgium; ReumaClinic, Genk, Belgium; Rheumatology, Maastricht University Medical Center, Maastricht, The Netherlands; Reuma Instituut, Hasselt, Belgium; Jessa Hospitals, Hasselt, Belgium

**Keywords:** Rheumatoid arthritis, Autoantibodies, Biomarker, Antibody isotype, UH-RA peptides

## Abstract

**Background:**

Recently, autoantibodies against novel UH-RA peptides (UH-RA.1 and UH-RA.21) were identified as candidate biomarkers for patients with rheumatoid arthritis (RA) who are seronegative for the current diagnostic markers rheumatoid factor and anticitrullinated protein antibodies. Previously, screening for anti-UH-RA autoantibodies was based on measuring the immunoglobulin (Ig) G response. We aimed to investigate whether measurement of other isotypes could improve the performance of diagnostic testing. In addition, assigning the isotype profile might provide valuable information on effector functions of the antibodies.

**Methods:**

The isotype profile of antibodies against UH-RA.1 and UH-RA.21 was studied. The IgG, IgM, and IgA classes, together with the 4 different IgG subclasses, were determined in 285 patients with RA, 88 rheumatic control subjects, and 90 healthy control subjects.

**Results:**

Anti-UH-RA.1 antibodies were primarily of the IgM isotype and twice as prevalent as IgG (IgG3-dominated) and IgA. RA sensitivity when testing for anti-UH-RA.1 IgM was shown to be higher than when testing for the IgG isotype: 18 % versus 9 % sensitivity when RA specificity was set to 90 %. Within antibodies against UH-RA.21, IgG and IgA were more common than IgM. Different anti-UH-RA.21 IgG subclasses were found, with the highest prevalence found for IgG2. Combined testing for IgG and IgA slightly increased RA sensitivity of UH-RA.21-specific antibody testing to 27 % compared with solely testing for IgG (23 %). Notably, a higher number of anti-UH-RA.21 antibody isotypes was related to increased levels of erythrocyte sedimentation rate. Finally, for both antibody responses, the full antibody isotype use was demonstrated in early and seronegative disease.

**Conclusions:**

The isotype distribution of anti-UH-RA.1 and anti-UH-RA.21 antibodies was successfully outlined, and, for antibodies against UH-RA.1, we found that isotype-specific testing might have implications for diagnostic testing. The exact mechanisms by which the different antibody isotypes act still have to be unraveled.

## Background

In the immunodiagnostics and pathogenicity of rheumatoid arthritis (RA), immunoglobulin (Ig) G is the most abundant antibody isotype in serum, and it is therefore most often used in clinical diagnostics. However, other Ig isotypes also have been proven to have utility. Testing for rheumatoid factor (RF), the first known antibody in RA, relies on the presence of IgM rather than IgG or IgA, although all isotypes are present before diagnosis and have been shown to be associated with disease severity and radiological outcome [1–3]. Also, the isotype repertoire has been investigated in the other antibody system currently included in RA diagnostics: anticitrullinated protein antibodies (ACPA). In those studies, in addition to IgG, IgM and IgA isotypes were frequently encountered [4–7]. Patients with RA present with more, different ACPA isotypes than their family members, indicating a difference in isotype use between health and disease [5]. Years before RA onset, ACPA of the IgG and IgA classes are present and predict the development of RA [8]. The ACPA isotype repertoire expands toward RA development and in the early course of the disease [4, 5, 9]. Besides the presence of ACPA, a broader range of ACPA isotypes predicts a higher risk for radiographic damage [10]. Measurement of isotype-specific autoantibodies can thus provide valuable information related to RA diagnosis and prognosis. The autoantibody isotypes might give information on the source of the antigen recognition, the major effector function involved, and the pathogenicity of the antibodies.

Previously, the presence of autoantibodies against UH-RA.1 and UH-RA.21—two novel peptides—was demonstrated in up to 23 % of seronegative patients with RA and one-third of patients with early RA [11, 12]. Testing for the novel autoantibodies (combined as UH-RA.PANEL2) was shown to reduce the serological gap by 9 %. On the one hand, antibodies against UH-RA.1 were associated with sustained disease-modifying antirheumatic drug (DMARD)-free remission. Anti-UH-RA.21 antibodies, on the other hand, were linked with worse outcomes, as associations with the presence of erosions, inflammation, and higher tender and swollen joint counts were found.

The primary aim of this study was to explore isotype use within anti-UH-RA.1 and anti-UH-RA.21 antibodies. Patients with RA were cross-sectionally tested for antibodies of IgG and all of its subclasses (IgG1–IgG4), IgM, and IgA. The presence of multiple isotypes within the antibody response might have implications for diagnostic and prognostic use. Moreover, the results of this study might provide insight into the biological role of the circulating autoantibodies, as Ig isotypes differ in their localization and biological properties.

## Methods

### Patient material

This study was approved by the medical ethics committee of Hasselt University (UH), and informed consent was obtained from all participants. Plasma samples of 285 patients with RA, 88 rheumatic control subjects (RC), and 90 healthy controls (HC) were used. Samples from patients with RA and RC subjects were collected between 2003 and 2012 in three Belgian rheumatology clinics. The diagnosis of RA was based on fulfillment of the 1987 criteria for RA [13], and samples were collected within the first year of diagnosis for 36 patients (early patients). HC were included if they were at least 18 years old and healthy, without any underlying chronic illness. Samples were stored in the University Biobank Limburg.

### Clinical data

The presence of erosions was registered as either present or absent. Additional clinical data retrieved from patients’ records were erythrocyte sedimentation rate (ESR), C-reactive protein, and the outcome of the Health Assessment Questionnaire together with disease activity measured using the Disease Activity Score in 28 joints, integrating measures of physical examination (tender and swollen joint counts), ESR, and patient self-assessment on a visual analogue scale. RF serology was evaluated in routine clinical laboratory testing with RF Latex reagent (upper limit of normal [ULN] 14 U/ml; Beckman Coulter, Brea, CA, USA), the RF-II cobas c system (ULN 14 U/ml; Roche, Vilvoorde, Belgium), or the SERODIA-RA test (Fujirebio Europe NV, Ghent, Belgium). ACPA testing was performed using the Phadia EliA cyclic citrullinated peptide (CCP) assay (CCP2; Thermo Scientific, Erembodegem, Belgium) or the QUANTA Lite CCP3 IgG enzyme-linked immunosorbent assay (ULN 19 units; INOVA Diagnostics Inc., San Diego, CA, USA).

### Peptide enzyme-linked immunosorbent assay

Plasma samples were tested for antibodies of the IgG, IgM, and IgA isotypes. Patients with positive test results for IgG were further tested for subclasses 1–4. Samples were tested for the specific peptide (P) (UH-RA.1: GLQEFGTREKRQEITTE and UH-RA.21: PGGFRGEFMLGKPDPKPEGKGLGSPYIE) and an irrelevant control peptide (C) (WTKTPDGNFQLGGTEP) according to a protocol described previously [11], with the minor modification of use of polystyrene flat-bottom microplates (Greiner Bio-One, Wemmel, Belgium) [11]. Furthermore, binding of plasma antibodies was detected using rabbit antihuman IgG secondary antibody (1:2000; Dako, Heverlee, Belgium); monoclonal mouse antihuman IgG1, IgG2, IgG3, or IgG4 secondary antibody (1:1000; Life Technologies, Ghent, Belgium); rabbit antihuman IgA secondary antibody (1:500; Dako; or goat antihuman IgM secondary antibody (1:5000; Sigma-Aldrich, Diegem, Belgium). All antibodies were conjugated to HRP. All samples were tested in duplicate within each experiment, and experiments were performed independently at least twice. A positive sample was included in each experiment to control for interassay variability.

### Statistical analyses

Antibody reactivity against UH-RA.1 and UH-RA.21 is expressed by the ratio of the optical density (OD) signal of specific peptide to the OD signal of control peptide. For each test, the cutoff value was set to 90 % specificity based on reactivity in the HC group. Proportions were compared by using the χ^2^ or Fisher’s exact test (expected count <5), while continuous data were compared between groups using the Mann-Whitney *U* test (MWU) for two groups or the Kruskal-Wallis test for more than two groups. Spearman’s ρ correlations were applied to study associations between continuous data. For all statistical tests, a *p* value <0.05 was considered statistically significant. Statistical analyses were performed using Prism version 5 (GraphPad software, La Jolla, CA, USA), IBM SPSS Statistics for Windows version 22.0 (IBM, Armonk, NY, USA), and JMP Pro version 11.2 (SAS Institute, Cary, NC, USA) software.

## Results

### Isotype distribution of anti-UH-RA.1 and anti-UH-RA.21 antibodies

The contribution of individual Ig classes of the IgG, IgM, and IgA types to total reactivity of anti-UH-RA.1 and anti-UH-RA.21 antibodies was investigated in 285 patients with RA, 88 RC, and 90 HC. The characteristics of our study population are provided in Table 1. Within the two antibody responses, the full isotype use was present.

**Table 1:** Characteristics of patients and controls tested for IgG, IgM, and IgA isotypes of antibodies against UH-RA.1 and UH-RA.21

Diagnosis	Number of subjects	Age, years	Female sex	Disease duration, years	RF-positive	ACPA-positive	RF/ACPA-positive
RA	285	60.1 ± 12.1	68 %	8.4 ± 7.8	56 %	48 %	63 %
RC	88	49.5 ± 11.6	42 %	10.2 ± 7.9^a^	NA	NA	NA
HC	90	38.7 ± 15.1	63 %	NA	NA	NA	NA

*Abbreviations: ACPA* anticitrullinated protein antibodies, *HC* healthy control subjects, *NA* not applicable or not available, *RA* patients with rheumatoid arthritis, *RC* rheumatic control subjects, *RF* rheumatoid factor

Data are mean ± SD unless otherwise indicated

^a^ Unknown for 17 RC

#### Anti-UH-RA.1 antibodies

**Fig. 1:**
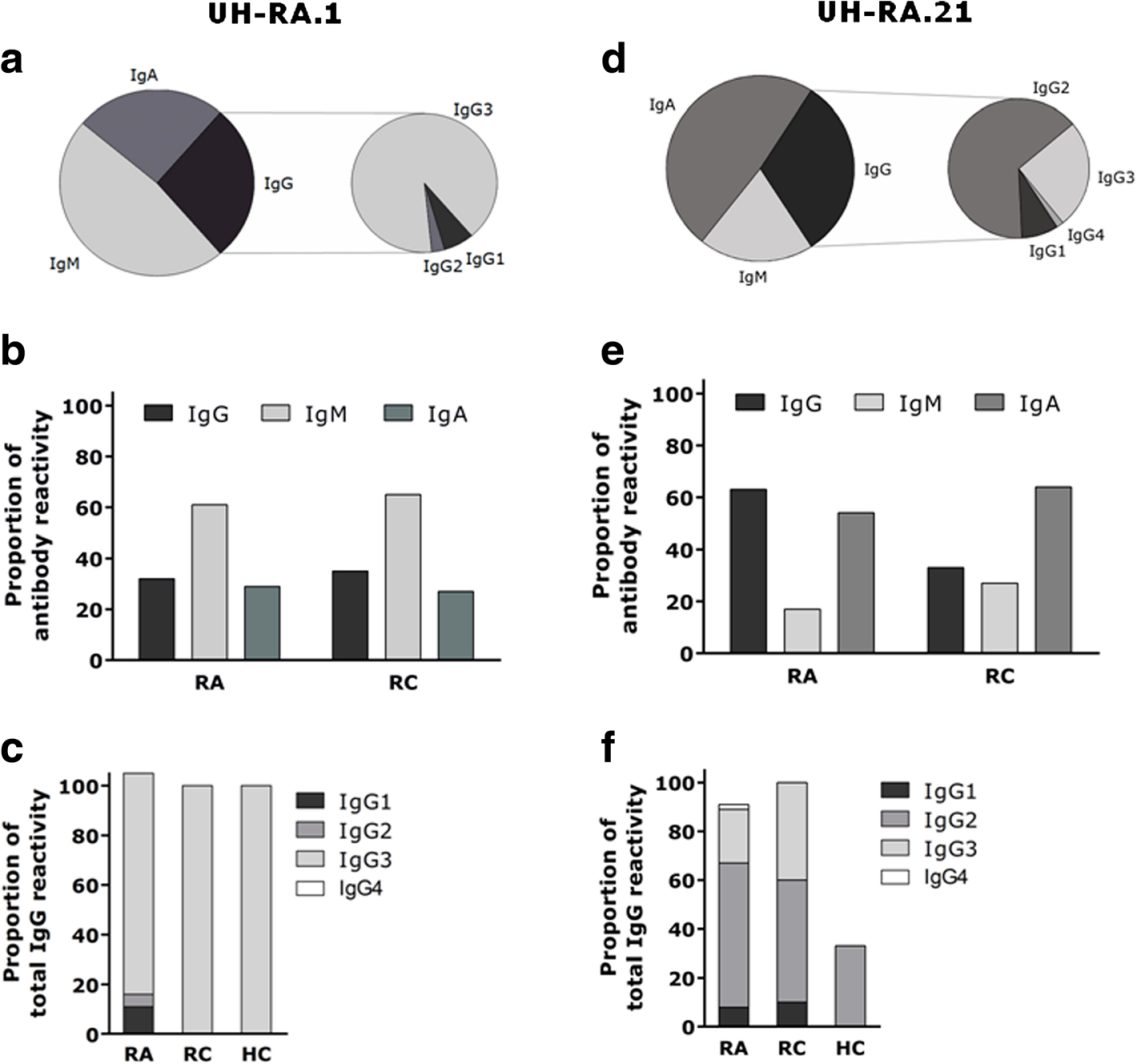
Prevalence of the IgG, IgM, and IgA (sub)classes within anti-UH-RA.1 antibodies and anti-UH-RA.21 antibodies. **a** Anti-UH-RA.1 and **d** anti-UH-RA.21 antibodies of the IgG, IgM, and IgA isotypes. **b** and **e** With cutoffs based on HC reactivity and set to 90 % RA specificity, the proportion of IgG, IgM, and IgA was similar in patients with RA and RC for antibodies against UH-RA.1 and UH-RA.21. **c** Subtyping of IgG-positive patients for IgG1–IgG4 pointed toward IgG3 as the dominant subclass within anti-UH-RA.1 antibodies in all patient and control groups. Anti-UH-RA.1-IgG1 and anti- UH-RA.1-IgG2 were RA-specific but less prevalent. No anti-UH-RA.1 antibodies of the IgG4 isotype were detected. **f** Regarding anti-UH-RA.21, IgG was represented mostly by IgG2, followed by IgG3 and IgG1. The latter two were less prevalent but not found in HC. Anti-UH-RA.21 antibodies of the IgG4 isotype were detected in one patient with RA. *Bars* represent the proportion of the respective isotype to the total antibody reactivity. Sums of individual proportions within the same patient group can exceed 100 % because patients can carry more than one (sub)class. *HC* healthy control subjects, *Ig* immunoglobulin, *RA* rheumatoid arthritis, *RC* rheumatic control subjects

Antibodies against UH-RA.1 were found in 130 individuals (82 RA, 26 RC, and 22 HC). Within these antibodies, IgM was most common, found in almost twice as many anti-UH-RA.1 antibody-positive patients compared with IgG and IgA (IgM 76/130 [58 %] versus IgG 44/130 [34 %] and IgA 40/130 [31 %]) (Fig. 1a). The distribution of the different isotypes was similar among patients with RA and RC (Fig. 1b). Twenty-nine IgG-positive individuals—of whom 19 were patients with RA, 6 were RC, and 4 were HC—were further subtyped for IgG1, IgG2, IgG3, and IgG4. This subtyping demonstrated that IgG reactivity was attributable mainly to the IgG3 subclass (Fig. 1a and c). IgG3 was present in 17 of 19 IgG-positive patients with RA and in all of the IgG-positive control subjects. IgG1 and IgG2 were RA-specific, but with a limited prevalence of 2 of 19 and 1 of 19, respectively (Fig. 1c). Anti-UH-RA.1 antibodies of the IgG4 subclass were not detected.

Up to 26 (20 %) of 130 of the anti-UH-RA.1 antibody-positive individuals harbored more than one antibody isotype (Table 2). When patients harbored two different antibody isotypes, mainly the combination of IgG with IgA (11/22) or IgA with IgM (9/22) was found, while the combination of IgG with IgM was less common (2/22). This pattern was also reflected by correlations between the levels of the different antibody isotypes: IgG levels were correlated with IgA levels (Spearman’s ρ = 0.254, *p* < 0.0001), and IgA levels were correlated with IgM levels (ρ = 0.269, *p* < 0.0001). No correlation was found between IgG and IgM (*p* = 0.984). Finally, due to the dominant role of IgG3, only two patients with RA carried more than one IgG subclass.

**Table 2:** Percentage of patients and controls positive for IgG, IgM, and IgA isotypes within antibodies against UH-RA.1 and UH-RA.21

	Anti-UH-RA.1 antibodies				Anti-UH-RA.21 antibodies			
Number of different isotypes present	Total (*n* = 463)	RA (*n* = 285)	RC (*n* = 88)	HC (*n* = 90)	Total (*n* = 463)	RA (*n* = 285)	RC (*n* = 88)	HC (*n* = 90)
0	333 (72)	203 (71)	62 (70)	68 (76)	305 (66)	182 (64)	55 (63)	68 (76)
1	104 (22)	66 (23)	21 (24)	17 (19)	116 (25)	72 (25)	26 (30)	18 (20)
2	22 (5)	14 (5)	3 (3)	5 (6)	36 (8)	27 (9)	6 (7)	3 (3)
3	4 (1)	2 (1)	2 (2)	0 (0)	6 (1)	4 (1)	1 (1)	1 (1)

*HC* healthy control subjects, *RA* patients with rheumatoid arthritis, *RC* rheumatic control subjects

The presence of anti-UH-RA.1 and -UH-RA.21 antibodies of the IgG, IgM, and IgA isotype was determined in 285 patients with RA, 88 RC, and 90 HC. Data are presented as absolute number (percent). Contingency analyses for individual isotypes or the number of isotypes did not show any significant differences between the three study populations

#### Anti-UH-RA.21 antibodies

Antibodies against UH-RA.21 were found in 158 individuals (103 RA, 33 RC, and 22 HC). In contrast to the antibody system against UH-RA.1, IgM was less redundant within antibodies against UH-RA.21: IgG and IgA were both twice as prevalent (IgM 35/158 [22 %] versus IgG 85/158 [54 %] and IgA 86/158 [54 %]) (Fig. 1d). Although not significant, the presence of IgG was higher in patients with RA than in RC, in whom IgM and IgA appeared slightly more often (Fig. 1e). IgG subtyping was performed in 67 IgG-positive patients (51 RA, 10 RC, and 6 HC). IgG2 was the most dominant isotype (30/51 [59 %]), followed by IgG3 (11/51 [22 %]) and IgG1 (4/51 [8 %]) (Fig. 1d and f). The latter two were present only in the RA and RC groups, whereas only IgG2 was found in the HC group. Anti-UH-RA.21 IgG4 antibodies were detected in one patient with RA (Fig. 1f).

For anti-UH-RA.21 antibodies, 36 of 158 individuals carried two different antibody isotypes (27 RA, 6 RC, and 3 HC) (Table 2). Six individuals carried three different antibody isotypes (4 RA, 1 RC, and 1 HC) (Table 2). Co-occurrence of two isotypes was dominated by IgG/IgA (26/36), while IgA/IgM (7/36) and IgG/IgM (3/36) were less frequently observed. IgA levels correlated with both IgG levels (ρ = 0.236, *p* < 0.0001) and IgM levels (ρ = 0.209, *p* < 0.0001), while IgG and IgM levels were not correlated (*p* = 0.247). The correlation between IgA and IgG levels was present only in the RA group.

### Implications of antibody isotype profiling for rheumatoid arthritis diagnostics

The diagnostic sensitivity of the anti-UH-RA.1 and anti-UH-RA.21 antibodies previously reported was established using a detection antibody directed against IgG, with only minimal cross-reactivity to other isotypes. However, since antibody reactivity is represented not only by IgG but also by IgM and IgA, we evaluated if isotype-specific testing could improve the diagnostic performance of anti-UH-RA.1 and anti-UH-RA.21 antibodies.

**Fig. 2:**
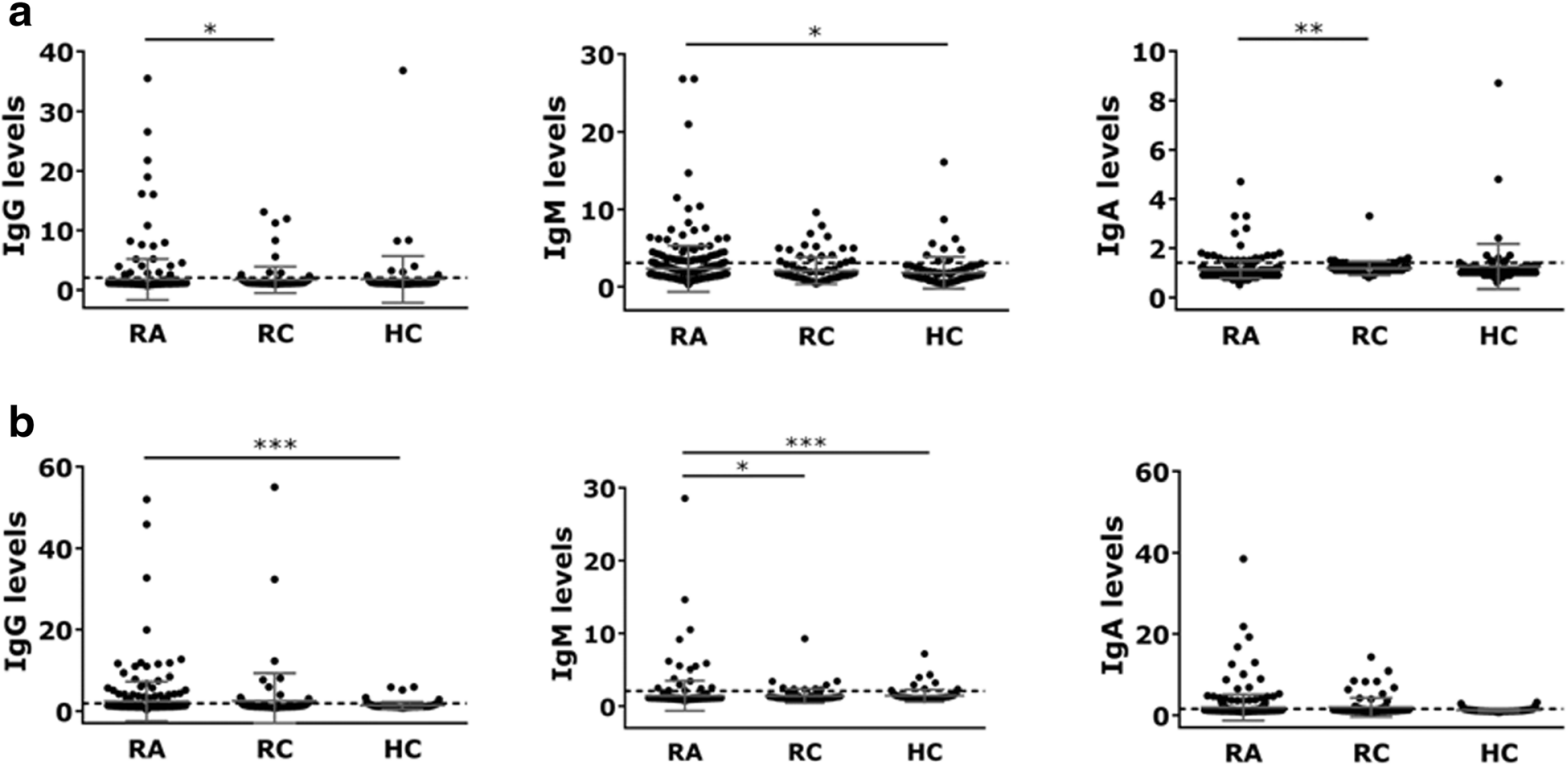
Levels of anti-UH-RA.1 (**a**) and anti-UH-RA.21 (**b**) antibody isotypes in patients with rheumatoid arthritis and control subjects. The *dashed lines* represent the cutoff value, set at 90 % based on reactivity in healthy controls. Antibody levels were compared by Kruskal-Wallis testing. *p < 0.05, **p < 0.01, ***p < 0.001. *HC* healthy control subjects, *Ig* immunoglobulin, *RA* patients with rheumatoid arthritis, *RC* rheumatic control subjects

The levels of isotype-specific antibodies against UH-RA.1 and UH-RA.21 are depicted in Fig. 2. Depending on the antibody isotype, anti-UH-RA.1 antibody levels were significantly higher in patients with RA than in RC (IgG, IgA) or HC (IgM) (Fig. 2a). Regarding UH-RA.21, patients with RA showed significantly higher levels of IgG and IgM compared with HC. IgM levels were also significantly higher in patients with RA than in RC (Fig. 2b).

**Fig. 3:**
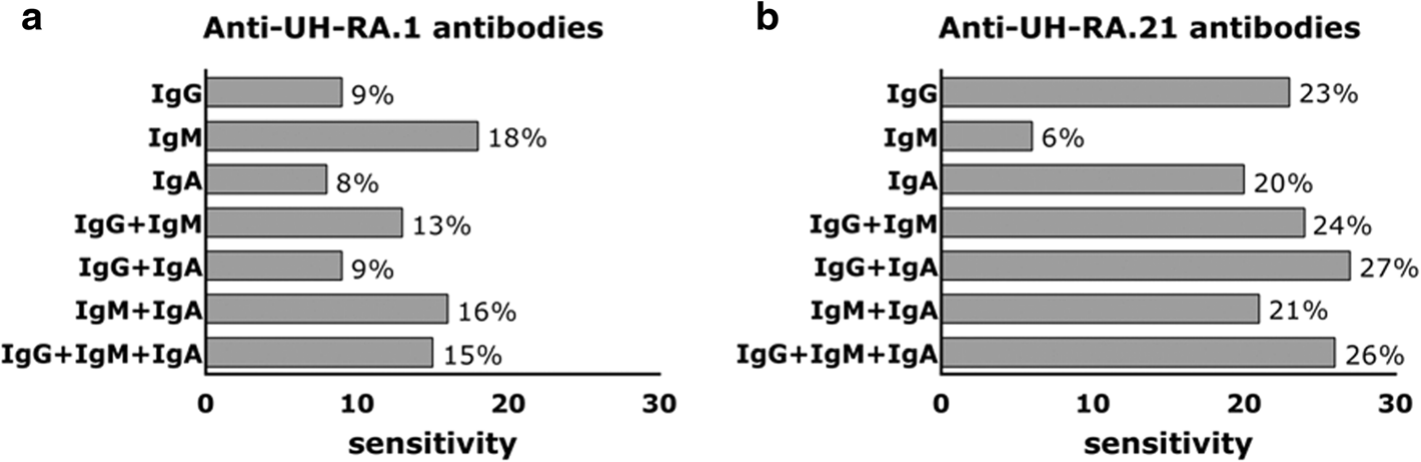
Sensitivity of isotype-specific testing for antibodies against UH-RA.1 (**a**) and UH-RA.21 (**b**) in patients with rheumatoid arthritis (RA), with an associated specificity of 90 %. Cutoff values were determined on the basis of reactivity in healthy controls, and specificity was set to 90 %. *Bars* represent the proportion of positive patients, and corresponding sensitivity for RA is provided. *Ig* immunoglobulin

Cutoff values based on reactivity in HC and set to 90 % specificity resulted in sensitivity values of 9 % for RA and 8 % for anti-UH-RA.1 IgG and IgA, respectively (Fig. 3a). The highest sensitivity for anti-UH-RA.1 antibody testing was achieved by testing for IgM (18 %). Even combining two or three antibody isotypes did not exceed this sensitivity observed for IgM. IgM together with IgG or IgA resulted in RA sensitivity of 13 % and 16 %, respectively. Because of the strong correlation between IgG and IgA, combined testing did not perform better than testing for both isotypes individually. The three antibody isotypes together ended up with a sensitivity of 15 %.

Within this study, testing for anti-UH-RA.21 IgG resulted in an RA sensitivity of 23 %, which was slightly improved by combined testing with IgM (24 %), IgA (27 %), or both (26 %) (Fig. 3b). Testing for anti-UH-RA.21 IgM or IgA yielded individual sensitivities of 6 % and 20 %, respectively, or a combined sensitivity of 21 %.

### Isotype-specific testing in early and seronegative rheumatoid arthritis

**Fig. 4:**
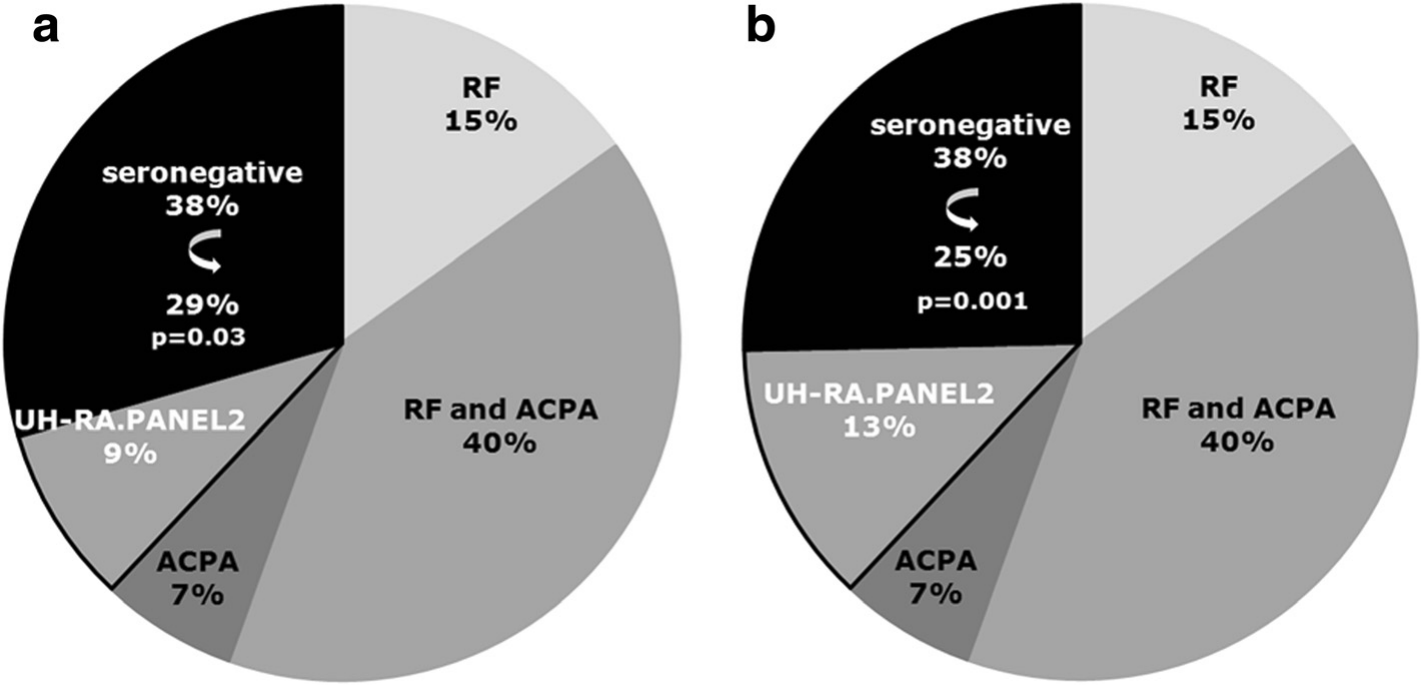
Improved diagnosis of seronegative patients with rheumatoid arthritis (RA) when testing for anti-UH-RA.1 immunoglobulin (Ig) M was added to the original IgG-specific testing for autoantibodies against UH-RA.1 and UH-RA.21. **a** Testing for novel autoantibodies against UH-RA.1 and UH-RA.21 (combined as UH-RA.PANEL2) was previously shown to reduce the serological gap by 9 %, based on IgG-specific testing. **b** Inclusion of antibodies against UH-RA.1 of the IgM isotype increases the sensitivity of the novel autoantibodies in patients with seronegative RA, improving the reduction of the serological gap from 9 % to 13 %. *ACPA* anticitrullinated protein antibodies, *RF* rheumatoid factor

The study population included 285 patients with RA, of whom 105 were RF−/ACPA−, 19 were RF−/ACPA+, 43 were RF+/ACPA−, and 118 were RF+/ACPA+. Within all serological subgroups, the full isotype use was found for antibodies against UH-RA.1 and UH-RA.21. Levels of anti-UH-RA.1 IgG, IgM, or IgA were not different between these serological subgroups. The sensitivities of the antibody isotypes within seronegative RA were 9 %, 20 %, and 9 % for IgG, IgM, and IgA, respectively, which were similar to the values for the total RA population. Notably, the reduction of the serological gap by 9 % following testing for IgG reactivity to UH-RA.1 and UH-RA.21 (combined as UH-RA.PANEL2), as reported recently (Fig. 4a) [12], was markedly improved to a reduction by 13 % after including anti-UH-RA.1 IgM testing in the panel (Fig. 4b).

For antibodies against UH-RA.21, no differences were observed in antibody levels between serological subgroups or sensitivities of individual isotype testing. The sensitivities of anti-UH-RA.21 IgG, IgM, or IgA in seronegative patients with RA were 24 %, 8 %, and 17 %, respectively.

Another diagnostically challenging subpopulation of patients with RA is patients in the early stage of the disease. This study population contained 36 patients with RA who were diagnosed not more than 1 year before sampling and therefore were classified as having early RA. While all antibody isotypes were found in early stages of the disease for both antibody systems (UH-RA.1 and UH-RA.21), the levels of IgG, IgM, and IgA isotypes were similar between the early and established RA subgroups.

### Prognostic information based on antibody (sub)class testing

**Fig. 5:**
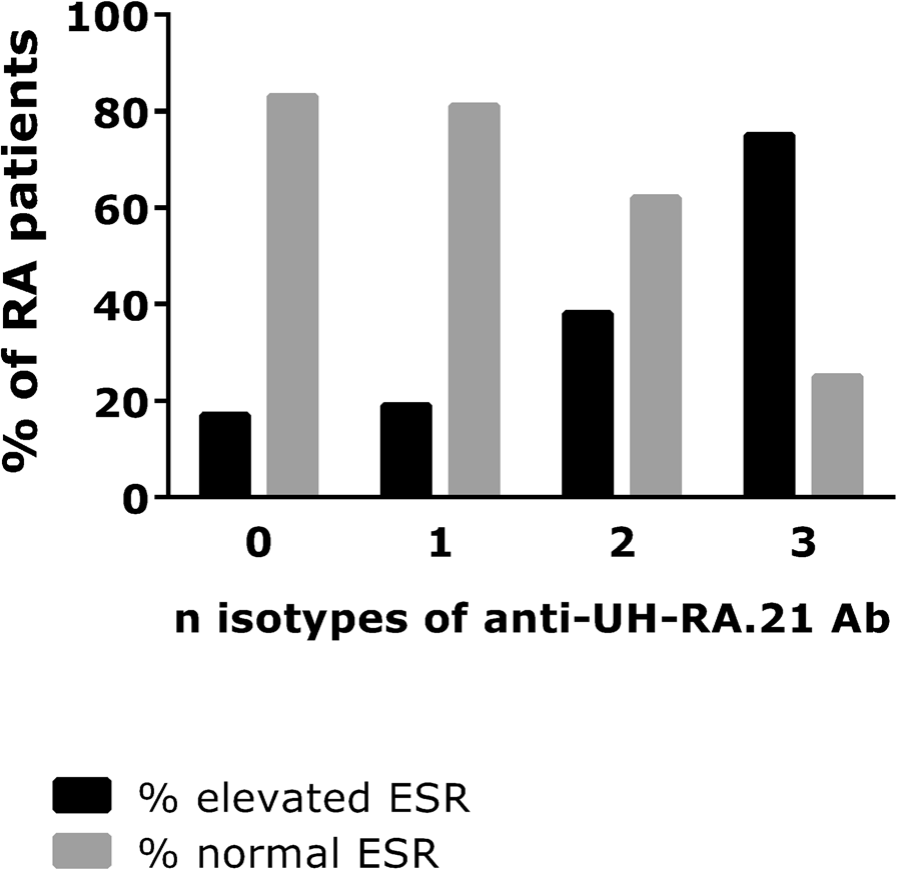
Percentage of patients with rheumatoid arthritis (RA) with normal and elevated erythrocyte sedimentation rate (ESR) in relation to the number of anti-UH-RA.21 antibody isotypes present (among immunoglobulin [Ig] G, M, and A). The presence of anti-UH-RA.21 antibodies of the IgG, IgM, and IgA isotypes was determined in 269 samples of patients with RA with data on ESR provided. An elevation of ESR was defined as >20 mm/h for men and >30 mm/h for women

Possible associations between antibody isotypes and clinical data were investigated to explore prognostic information based on isotype distribution. Within this study, a link was found between smoking and the presence of anti-UH-RA.1 antibodies of the IgA class: IgA was found in 4 of 16 smokers and 0 of 77 nonsmokers (*p* = 0.001 by Fisher’s exact test). No other prognostic information could be deduced from isotype-specific testing for anti-UH-RA.1 antibodies. Regarding antibodies against UH-RA.21, an association was found between the presence of erosions and levels of IgG (*p* = 0.028 by MWU) or IgG2 (*p* = 0.033 by MWU). For both (sub)classes, associations were observed for ESR (IgG: *p* = 0.010 by MWU and *p* = 0.045 by χ^2^; IgG2: *p* < 0.0001 by χ^2^). Additionally, ESR was associated with a positive test result for anti-UH-RA.21 IgM (*p* = 0.004 by χ^2^). Finally, ESR levels were also higher when a higher number of different anti-UH-RA.21 antibody isotypes were present, as shown in Fig. 5 (χ^2^ = 13.20, *p* = 0.004).

## Discussion

In this study, we report the contribution of individual Ig classes of the IgG, IgM, and IgA types to total reactivity of novel autoantibodies against UH-RA.1 and UH-RA.21. Both antibody systems were represented by the full isotype repertoire, and the isotype profile was similar in patients with RA and RC. The major difference in isotype distribution between the two antibody systems was the contribution of IgM. For antibodies against UH-RA.1, IgM accounted for half of the reactivity, whereas for anti-UH-RA.21 antibodies the other two isotypes were twice as prevalent. IgM is the first antibody produced during the primary humoral immune response [14]. The presence of IgM is therefore associated with recent antigen exposure. If the antigenic stimulus persists, the μ chain is changed to γ (IgG) or α (IgA) [15]. The presence of the latter isotypes thus points toward a secondary antibody response. Because the presence of IgG is typical of a persistent immune response and because of its relative abundance, IgG is often the first choice in diagnostic testing for chronic conditions. However, as the presence of IgM is indicative of recent antigen exposure, we can presume that the antibody response against UH-RA.1 (and, to a lesser extent, UH-RA.21) is marked by an ongoing immune response and a continuous (re)activation of the immune system. How IgM production is sustained in the presence of IgG against the same antigen is not fully understood, but similar observations have been reported for ACPA [16]. IgA, then, is the key component of the humoral response in mucosal tissue such as the lungs or the gut [17]. Its presence in the mucosa of the lungs to protect the epithelial surface might explain the higher participation of anti-UH-RA.1 IgA in smokers than in nonsmokers as has also been observed for RF and ACPA. Patients with pre-RA who were smokers were significantly more often IgA RF-positive [18]. Furthermore, IgA ACPA appeared earlier in smokers than in nonsmokers [5, 7, 8].

Regarding the IgG subclasses, the majority of anti-UH-RA.1 antibody reactivity was attributable to IgG3, the most proinflammatory and pathogenic subclass because it is a potent activator of the complement cascade [19, 20]. This role is shared with IgG1, also found within the anti-UH-RA.1 antibody response. The dominance of the IgG3 subclass in the anti-UH-RA.1 antibody system seems not in line with previous findings of an association between the presence of the antibodies and the achievement of sustained DMARD-free remission [12]. However, IgG antibodies can act in anti-inflammatory ways as well through the engagement of type II Fc receptors (FcRII) rather than FcRI [21]. This is mediated by the glycan core structure of IgG, and modifications such as galactosylation and sialylation have been shown to shift the activity from pro- to anti-inflammatory [22–26]. Sialylated IgG-Fc, for instance, upregulates the inhibitory receptor FcγRIIB, increasing the activation threshold of innate effector cells to immune complexes [27].

For IgG subclasses directed against UH-RA.21, IgG2 was the most abundant subclass, followed by IgG3 and IgG1. IgG2 is considered less pathogenic than IgG3 and IgG1, and so far the IgG2 isotype is less comprehensively understood. Further characterization of the biological properties (e.g., glycan modifications) of the anti-UH-RA.1 and anti-UH-RA.21 antibody isotypes will clarify the significance of the isotypes in the pathophysiology of RA.

The use of multiple antibody isotypes raised the question whether isotype-specific testing could add value to the diagnostic performance of the antibodies. Up to now, antibody reactivity against UH-RA.1 and UH-RA.21 has been measured using an anti-IgG detection antibody. In the present study, isotype-specific testing for anti-UH-RA.1 antibodies suggested an improvement by testing for IgM rather than IgG, as the sensitivity in patients with RA was twice as high (18 % versus 9 %) when RA specificity was set to 90 %. Also, the levels of anti-UH-RA.1 IgM were significantly higher in patients with RA than in RC or HC. Although IgG1 and IgG2 were RA-specific compared with RC and HC, their prevalence was too low to consider them for isotype-specific diagnostic testing. For anti-UH-RA.21 antibody testing, the sensitivity was shown to increase when IgA was added to the current IgG detection. Importantly, the full antibody isotype use was already present in early stages of the disease. Furthermore, the full antibody isotype use was detected in seronegative patients with RA. These findings further support the promising role of the antibody systems in the diagnosis of patients with early and seronegative RA.

## Conclusions

We examined the isotype distribution of antibodies against UH-RA.1 and UH-RA.21. Since effector functions differ between antibody classes and subclasses, the study of the isotype profile is important to understanding the pathophysiological role of the antibody systems. At present, we can only speculate about the exact mechanisms by which anti-UH-RA.1 and anti-UH-RA.21 antibodies work, but we have outlined the isotype profile for both antibodies. The exact mechanisms by which the isotypes act need further investigation. The main finding of this work is that full antibody isotype use in early and seronegative RA was demonstrated. The impact of the full use of the antibody isotype repertoire was evaluated for diagnostic application, and, interestingly, the sensitivity of anti-UH-RA.1 antibodies was shown to increase considerably when we measured IgM instead of IgG.

## Abbreviations

ACPA, anticitrullinated protein antibodies; CCP, cyclic citrullinated peptide; DMARD, disease-modifying antirheumatic drug; ESR, erythrocyte sedimentation rate; FcR, Fc receptor; HC, healthy control subjects; Ig, immunoglobulin; MWU, Mann-Whitney *U* test; NA, not applicable or not available; OD, optical density; RA, rheumatoid arthritis; RC, rheumatic control subjects; RF, rheumatoid factor; UH, Hasselt University; ULN, upper limit of normal
